# What is the role of portion control in weight management?

**DOI:** 10.1038/ijo.2014.82

**Published:** 2014-07-25

**Authors:** B J Rolls

**Affiliations:** 1 Department of Nutritional Sciences, The Pennsylvania State University, University Park, PA, USA

## Abstract

Systematic studies have shown that providing individuals with larger portions of foods and beverages leads to substantial increases in energy intake. The effect is sustained over weeks, supporting the possibility that large portions have a role in the development of obesity. The challenge is to find strategies to effectively manage the effects of portion size. One approach involves teaching people to select appropriate portions and to use tools that facilitate portion control. Although tools such as portion-control plates have been shown in several randomized trials to improve weight loss, limited data are available on whether education and tools lead to long-term changes in eating behavior and body weight. Another approach is to use preportioned foods (PPFs) to add structure to meals and minimize decisions about the amount of food to eat. A number of randomized controlled trials have demonstrated the efficacy of both liquid meal replacements and solid PPFs for weight loss and weight loss maintenance, but it is not known if they lead to better understanding of appropriate portions. Although portion control is important for weight management, urging people simply to ‘eat less' of all foods may not be the best approach as high-energy-dense foods disproportionately increase energy intake compared with those lower in energy density. A more effective strategy may be to encourage people to increase the proportion of foods low in energy density in their diets while limiting portions of high-energy-dense foods. If people lower the energy density of their diet, they can eat satisfying portions while managing their body weight.

## Introduction

In recent decades, increases in portion size have occurred in parallel with the rise in the prevalence of obesity.^1, 2, 3^ Furthermore, multiple controlled studies have shown that providing individuals with larger portions of foods and beverages leads to substantial and sustained increases in energy intake.^4, 5, 6, 7, 8, 9^ These data suggest that the availability of large portions can override the regulation of energy balance and can have persistent effects that could lead to obesity. In 2010, the Dietary Guidelines Advisory Committee found that ‘strong evidence documents a positive relationship between portion size and body weight'.^10^ That report and other recent policy documents on weight management stress the importance of portion control to help limit energy intake.

The goal of this review is to extend previous reviews on the evidence linking portion size to energy intake and body weight status in adults^4,11,12^ and to discuss strategies to moderate the influence on intake of large portions of energy-dense foods. Consideration will also be given to strategies to use the robust effects of portion size positively to increase intake of nutrient-dense foods. Finally, the types of portion control strategies that have been applied to weight management will be considered.

## Studies linking portion size and intake

### Early studies

The first known study linking portion size and intake came from Siegel^13^ in 1957 who found that people have a ‘completion compulsion' or tendency to eat entire servings or units of food. In that brief report, few data were presented and there were no statistics, but there were memorable observations such as: ‘In cookie consumption, compulsion is marked enough to stimulate a chuckle. On only one occasion was a fraction of a cookie left behind.' Over the next 30 years, attempts to establish a significant link between portion size and consumption were disappointing. In one study, normal-weight subjects, offered either one sandwich at a time (with more available in a refrigerator) or three sandwiches together, ate an average of two sandwiches in both conditions.^14^ In 1986, Edelman *et al.*^15^ tested the effect of varying the portion of lasagna available in a cafeteria at the military facility in Natick, Massachusetts.^15^ Although the portion offered varied significantly (255 vs 426 g), no effect of portion size was found. It is not clear why portion size failed to affect intake in these studies, but it may have been that participants could have more helpings than were initially served. Ten years later, Wansink^16^ showed that the package or container size of a variety of foods (spaghetti, chicken and chocolate candies) affected the amount people took, but intake was not assessed. In a later relatively uncontrolled study conducted in a movie theater, consumers were found to eat more from a large bucket of popcorn than from one half the size.^17^ Thus, until recently, there was little experimental evidence showing a direct effect of portion size on intake.

Meanwhile, data were accumulating that portion sizes of many foods and beverages had increased substantially.^2^ Furthermore, nationally representative survey data from the United States indicated that as portions increased in size, people reported that they had been consuming larger portions. Between 1977 and 1996, the amount consumed at an eating occasion increased for a wide range of foods.^1,3^ In a seminal paper published in 2002 with the title ‘The contribution of expanding portion sizes to the US obesity epidemic', Young and Nestle^2^ drew a parallel between the increases in portion sizes of restaurant foods, grocery products and recipes in cookbooks and the rise in the prevalence of obesity. Although the overlap in the rise in portion size and obesity prevalence was compelling, more direct evidence that portion size affects intake was needed.

### Amorphous foods

A crucial step in relating the expansion of portion sizes to increased obesity was to determine systematically whether portion size affects energy intake. Thus investigators in my lab undertook a series of investigations to test how varying the portion size of different types of foods and beverages affects energy intake. Macaroni and cheese was studied first as the portion size of such amorphous foods is particularly difficult to judge, especially when the portions are large.^18^ When subjects were offered four portions of macaroni and cheese on different days, intake of largest portion (1000 g) was 30% (162 kcal) greater than of the smallest portion (500 g).^5^ The effect was seen both when we determined the portion on the plate and when the participants served themselves from bowls containing different portions. The results were not affected by subject characteristics, including sex, body mass index or concern about food intake or body weight. Many participants appeared unaware of variations in portion size in that less than half of them reported noticing that there were differences in the portions served, and ratings of hunger and fullness were similar after eating despite differences in intake.^5^

### Unit foods and packages

We conducted further studies to test whether intake can be influenced by the portion size of other types of foods such as those with clearly defined shapes or units. It is possible that with such foods people would be less susceptible to the effect of portion size as they may have learned how many units of a particular size are appropriate. We tested this by offering on four different days deli-style sandwiches that varied in size: 6, 8, 10, and 12 inches.^7^ We found a systematic and significant effect of portion size on intake for both men and women. When served the 12-inch sandwich compared with the 6-inch sandwich, women consumed 31% more energy and men consumed 56% more energy. Even with foods having a clearly defined unit size bigger portions lead to greater energy intake.

In 1996, Wansink^16^ demonstrated that package size influenced how much food people took; however, controlled studies were needed to test the influence of package size on how much is eaten. Therefore, on five occasions we served men and women an afternoon snack that consisted of 28, 42, 85, 128 or 170 g of potato chips in a plain, unlabeled foil bag.^6^ Participants ate directly from the bag so that they had few visual cues to guide consumption. The results showed that portion size had a significant effect on snack intake for both men and women. For example, when served the 170-g package, women ate 18% more and men ate 37% more than when served the 85-g package. As subjects increased their snack intake with increasing package size, they reported feeling fuller; however, they did not adjust their intake at the subsequent dinner to compensate for the increased energy intake. Thus, bigger portions of a prepackaged snack increased energy intake in the short term. This effect may be related to the tendency to eat whole units of food as proposed by Siegel^13^ in 1957 and others more recently.^19,20^

As package size can have a significant influence on how much is consumed, several studies have tested whether portion-controlled packaging can be used to help moderate intake of energy-dense foods. The results have been mixed, with some studies indicating that small packages such as 100-calorie snacks are associated with reduced consumption,^21^ especially in people who are overweight.^22^ Other studies indicate that this approach can lead to a lapse in self-control and increased consumption, particularly in individuals trying to restrain their intake.^23,24^ The marketing of small packages of energy-dense foods as appropriate for weight management may give dieters license to relax their control over energy intake. These findings emphasize the complexity of eating behavior and the need for more studies to determine how to translate basic research on portion control to the consumer world.

### Beverages

Although beverages are thought to present a particular challenge to the maintenance of energy balance, in that liquid energy may not be well-sensed by mechanisms that regulate food intake,^25^ relatively little is known about the impact of variations in beverage portion size. We tested this by serving different beverages (cola, diet cola or water) in two portions (360 g per 12 fl. oz. or 540 g per 18 fl. oz.) along with a standard lunch on six occasions and found that food intake did not differ across conditions, but beverage intake varied with portion size. When served the larger portion of regular soda, women increased their beverage intake by 10% and men by 26%. The energy from the soda added to that from the foods consumed during the meal. Thus serving large portions of caloric beverages at a meal can significantly increase overall energy intake.^26^

It is not clear how to discourage consumption of oversized sugar-sweetened beverages. Two studies conducted in the Netherlands in either a cinema^27^ or a fast food restaurant^28^ found that providing labels about the portion size or energy content did little to influence the size of soda chosen by customers. Legislation has been proposed that would limit the size of soda offered to customers in some restaurants; however, opinions are mixed as to how such restrictions would affect intake.^29,30^

## Restaurant portions

It is surprising that in studies conducted in a controlled laboratory setting where the main focus was on food and eating, many participants appeared to be unaware of the changes in the portions offered and how such changes affected the amount they ate. It seems likely that when individuals are in situations where there are more distractions, such as when eating out, they would be even less aware of portion size. We tested how increasing the portion size of a popular entrée (the main dish at a meal) would affect intake in a restaurant setting.^31^ Customers who were served 50% more of a pasta dish ate 43% more than those served a standard portion. A survey filled out by the customers showed no difference in ratings of the appropriateness of the two portions. Thus, in restaurants as well as in the laboratory, portion size can have a significant impact on energy intake, and consumers may be unaware of this effect.

Many restaurants provide portions with more calories than are needed by their customers and this may partly explain why eating out has been found to be associated with overweight and obesity.^32,33^ In a survey of >1000 adults, 69% indicated that, when dining out, they finish their entrées all or most of the time. Of those adults, 30% reported that they would have been satisfied with a smaller portion.^34^ Indeed, a recent study conducted in a Chinese fast-food restaurant showed that 14–33% of customers, when offered, chose to take a smaller side dish even if there was no price incentive to do so.^35^ The investigators also found that labeling the calorie content of menu items did not affect what customers ordered. It is important to find effective ways to communicate with customers what constitutes an appropriate portion. Clearly patrons need to understand their own calorie needs if menu labeling is to help them maintain energy balance.^36^ In addition to the potential impact of labels on consumers' choices, disclosure of the calorie content of menu items could act as an incentive for restaurants to modify the foods offered so that it is easier for customers to make appropriate choices.^37^

Improvements in menu options depend to some extent on chefs' opinions of the portion sizes and calorie content of the items they serve. In a survey of 300 chefs, they reported the portions served were primarily influenced by the presentation of foods, food cost and customer expectations, while the calorie content had relatively little effect.^38^ Although most chefs thought that the amount of food they serve influences how much patrons consume, their opinions were mixed about whether it is their responsibility or the customer's to eat an appropriate amount when served a large portion. In another survey of 432 chefs, nearly all thought that calories in menu items could be reduced by 10–25% without customers noticing, but they noted low consumer demand as the primary barrier to making such changes.^39^ With both chefs and customers thinking of oversized portions as appropriate, it is critical to find strategies that will help consumers avoid excess energy intake associated with oversized portions of energy-dense foods when eating out.

## Persistent effects of portion size

A number of studies show a clear effect of portion size on intake at a single eating occasion, but what happens when large portions are continually available? Will people keep overeating or will compensatory mechanisms sense the accumulating excess energy and limit intake? We first tested this by increasing the portion size of all available foods and beverages by 50% or 100% over 2 consecutive days.^9^ Effects persisted over the 2 days such that increasing portions by 50% increased participants' daily energy intake by 16% and increasing portions by 100% increased intake by 26%. Another study conducted in a residential facility in Northern Ireland found a significant and sustained increase in energy intake over 4 days when larger portions were served.^40^ An even longer study showed that increasing portions of all available foods and beverages by 50% over 11 days resulted in a mean increase in daily energy intake of 423 calories.^8^ This increase in daily energy intake was sustained over the 11-day period leading to a cumulative increase in intake of 4636 kcal. Additionally, when employees of a medical center were provided with free box lunches for 2 months, 1 month for each of two portion sizes (767 vs 1528 kcal), energy intake at lunch was 332 kcal higher with the larger portions. This resulted in an increase in daily energy intake of 278 kcal, and there was no significant compensation for this increase over the month.^41^

Thus the availability of large portions over a number of days is associated with a sustained increase in energy intake. The continuous availability of large portions can override biological compensatory responses associated with excess energy consumption, supporting the possibility that large portions of energy-dense foods have a role in the development of obesity.

## Leveraging the robust effect of portion size to improve diets

As the effect of portion size is robust and sustained, it is possible that it could be used beneficially to increase intake of nutritious, low-energy-dense foods, such as vegetables. Recent dietary advice relies on this premise. The 2010 Dietary Guidelines for Americans,^10^ which focus on obesity prevention, urge people to increase the proportion of vegetables and fruits served at a meal. Evidence to support this advice comes from a study in which increasing the vegetable portion on the plate by substituting it for the meat and grain significantly increased vegetable intake and reduced energy intake at the meal.^42^ Other studies show that increasing the portion of vegetables served at the start of a meal increases vegetable intake^4,43^ and can decrease energy intake at a meal. Such findings support the suggestion that variations in portion size can be used beneficially to influence the types and amounts of foods consumed at a meal. Although there are no data on the effects of such strategic manipulations of portion size on body weight, advice to simply eat more vegetables and fruits has not been found to lower body weight unless dietary energy density was reduced.^44, 45, 46, 47^

## Is ‘eat less' the best message?

An examination of weight-control strategies reported by obese participants trying to lose weight in the 2001–2006 National Health and Nutrition Examination survey showed that the most prevalent approach was to eat less food.^48^ This fits with the 2010 Dietary Guidelines for Americans,^10^ which recommend that people eat less overall and avoid oversized portions. Although the most obvious strategy to counter the effects of large portions is simply to eat less of everything, this may be hard for consumers to adopt and sustain. Nor is it necessary for people to eat less food overall to manage their weight.^49^ If people substitute vegetables and fruits and other nutrient-rich, low-energy-dense foods for foods higher in energy density, they will be able to eat more food for a given number of calories. Advice about appropriate portions should emphasize to consumers that they can consume satisfying amounts of food if they lower the energy density of their diet. For example, if people consume their usual amount of food, even modest changes in energy density can have a significant impact on daily energy intake. On a typical day, an adult might consume 1200 g of food with an overall energy density of 1.8 kcal g^−1^, giving an energy intake of 2160 kcal. If the average energy density of the diet was decreased by 0.1 kcal g^−1^ while the same weight of food was consumed, then the individual would ingest 2040 kcal. Thus a small change in the overall energy density of the diet would reduce energy intake by 120 kcal per day, and people could continue to eat their usual amount of food. Numerous studies show that individuals have a strong tendency to eat a consistent weight of food over a day and therefore strategies that rely on maintaining smaller portions of all foods are unlikely to be optimal or sustainable.^50, 51, 52^

At every eating occasion, energy intake can be influenced by both the portions available and their energy density. Several studies show how energy density and portion size combine to influence energy intake.^4^ In the first study, a lunch entrée was served at two energy density levels in three different portion sizes on different days.^53^ Subjects consumed 56% more energy when served the largest portion of the high-energy-dense entrée, compared with when served the smallest portion of the low-energy-dense entrée. The effects of energy density and portion size acted independently and added together to influence energy intake. To determine whether the combined effect of energy density and portion size persists beyond a single meal,^54^ participants in another study were provided with a variety of popular, commercially available foods for all their meals over 2 consecutive days on four different occasions. The same menus were served during each 2-day session, but all foods were varied in both energy density and portion size between a standard level and a reduced level (75% of the standard). Again, the effects of energy density and portion size combined so that energy intake was the greatest with the large portions of high-energy-dense foods and was 800 kcal lower (32%) on each of the 2 test days with the reduced portions of the lower-energy-dense foods. These findings suggest that food providers could use the combined effects of portion size and energy density to develop foods that will help consumers reduce their energy intake.

Recommendations related to appropriate portions need to consider the energy density of the food to be consumed. The combined influence of energy density and portion size on energy intake suggests that simply telling people to eat less of everything may not be the most effective message. Instead, messages should be focused on encouraging people to consume a greater proportion of their diet from foods lower in energy density while limiting portions of high-energy-dense foods.

## Portion size and weight management

Although numerous studies show that portion size affects energy intake, few have demonstrated a link between portion size and body weight or adiposity in adults. A small experimental study from the Netherlands found that obese women consumed larger portions of high-energy-dense foods and smaller portions of low-energy-dense foods, as compared with non-obese women.^55^ Another study using data from the National Diet and Nutrition Survey of British adults found little association between obesity status and reported portion sizes of specific food groups. The authors speculated that the high level of under-reporting may have masked associations.^56^ Clearly more studies of both experimental and population-based data are needed to understand relationships between portion size, energy density and weight status. Nevertheless, there is an urgent need for evidence-based strategies to help consumers limit the impact on intake of large portions of energy-dense foods. Reports on weight management from professional organizations and government agencies stress the importance of portion control to help limit energy intake but also emphasize the need for further research. For example, The Academy of Nutrition and Dietetics evaluated the evidence and found:

“Although the concept of portion control is universal in most weight management programs, the overall strength of the evidence for portion control to reduce energy intake is graded as fair. More research is needed to determine the effectiveness of specific portion control strategies on body weight regulation”.^57^

In 2006, The Centers for Disease Control and Prevention, in a Research to Practice Report, recognized that portion size can influence weight management but pointed out gaps in research. For example, the report found no published intervention studies that focused on how training people to recognize appropriate serving sizes affected their eating behavior and body weight.^58^

The assessment of the effectiveness of portion-control strategies as only fair, as well as the identification of major gaps in research, is surprising as portion control is a component of many weight-management programs. In the rest of this review, studies that have been conducted relevant to portion control and weight management and their implications will be discussed.

## Portion-control tools

An obvious strategy to counter ‘portion distortion' is to educate people to recognize how much they should eat of different types of foods and beverages at meals and snacks. A number of instructional materials related to portion size are available from health organizations.^59, 60, 61, 62^ Individuals receiving dietary counseling are often taught to use measuring tools (scales, cups and spoons and photographs) and these have been shown to improve estimation accuracy.^63^ However, there have been only a few randomized controlled trials (RCTs) examining whether portion-control tools facilitate weight management. Two 6-month RCTs tested the utility of plates specifically designed to indicate appropriate portions. In one, the results showed that diabetics using such a portion-control plate for 6 months lost significantly more weight than those who received usual care (1.8% vs 0.1% of body weight).^64^ Results of the other RCT were similar in that adding a portion-control plate to a weight loss intervention facilitated weight loss (2.4% vs 0.5% at 3 months).^65^ These trials, while showing that portion-control tools can be a useful addition to weight management programs, did not test whether using the portion-control plate can lead to better understanding of appropriate portions. It is also not clear whether such tools lead to persistent changes in food selection or whether they promote the maintenance of weight loss.

A challenge is to match tools to an individual's needs so they will either continue to be used or they will have a sustained impact on understanding of how to make portion choices. This will perhaps be facilitated as new electronic tools become more widely used. These give guidance about portion size based on information people record at the time of eating, including photographs of their meals and snacks. Such immediate feedback could help consumers understand if they are eating appropriate amounts. It is hoped that developers of such products will conduct RCTs to test the effectiveness for both weight loss and weight maintenance.

## Portion-controlled foods and beverages

Another portion-control strategy that individuals can utilize is to structure their food environment so that exposure to large portions of energy-dense foods is limited during one or more eating occasions in a day. This can be achieved with preportioned foods (PPFs) such as entrées, snack foods or liquid meals that are packaged to provide appropriate portions for a variety of eating occasions. Existing evidence suggests that just as the availability of large portions leads to overconsumption, portion-controlled foods can help to limit energy intake and promote weight loss.

### Liquid meal replacements

A number of studies have tested the utility of liquid meal replacements for weight loss. A systematic evaluation of RCTs found that substituting a liquid meal replacement for one or two meals per day improved weight loss and compliance over 1 year compared with lifestyle change programs emphasizing calorie reduction.^66,67^ Of particular interest is that of the 276 publications identified as relevant in this evaluation, only six met the criteria for a well-controlled RCT. The experimental design makes many of the studies on the efficacy of meal replacements difficult to interpret as few were designed to determine whether meal replacements are associated with greater weight loss than a self-selected diet of regular foods.^68^ Nevertheless, there is general consensus that meal replacements represent one of the most effective tools for weight loss.^51^ The European Food Safety Authority, which evaluates and substantiates health claims for commercial products, considers that ‘meal replacement for weight control' is sufficiently characterized in relation to the claimed effects.^69^ They conclude ‘that a cause and effect relationship has been established between the consumption of meal replacements in substitution of regular meals in the context of energy restricted diets and reduction in body weight.' A similar claim was approved for the maintenance of body weight after weight loss. The strength of the evidence and the ease of use have encouraged the inclusion of meal replacements in large-scale weight management trials such as Look AHEAD.^70^ Results from that trial have confirmed their utility in that at the end of the first year the amount of weight lost was related to the number of meal replacement products used.^67,70^

### Pre-portioned foods (PPFs)

Although there is an indication from studies conducted in Germany that consumption of liquid meal replacements can facilitate maintenance of weight loss for periods of up to 4 years,^71^ it is possible that for many consumers they may not retain the appeal needed for continued use.^72^ An alternative approach is to consume conventional foods that have been packaged in amounts appropriate for single meals or snacks or PPFs. Although there have been relatively few RCTs specifically testing the efficacy and effectiveness of PPFs for long-term weight management, PPFs may provide an even better tool than liquid meal replacements. Several short-term studies have found that solid meal replacements (bars) enhanced satiety more than isocaloric liquid meal replacements (shakes).^73,74^ The wide variety of commercially available PPFs could also help to make their continued use more sustainable than the relatively monotonous liquid meal replacements.

Some of our understanding of the utility of solid PPFs for weight management has stemmed from studies aimed at determining which strategies most effectively help patients comply with dietary interventions.^75,76^ In an 18-month RCT, participants were assigned to one of the four groups: a standard behavioral treatment, a behavioral treatment supplemented with financial incentives for weight loss, food provision, or a combination of food provision and incentives. The food that was provided consisted of preportioned conventional foods appropriate for five breakfasts and five dinners each week over the 18 months. Weight loss in the two groups provided with food was significantly better than the other groups at 6, 12 and 18 months.^75,76^ In another multicenter year-long RCT, patients were assigned either to a prepackaged, nutritionally complete, prepared meal plan that provided most of their food or to a macronutrient equivalent usual-care diet. The prepared meal plan promoted and sustained long-term weight loss such that at 1 year that group had lost 5.8 kg compared with a loss of 1.7 kg in the usual-care group.^77^

Two RCTs have been conducted specifically to test the efficacy for weight loss by providing portion-controlled entrées.^78,79^ When men and women were provided with two frozen, portion-controlled entrées daily for 8 weeks, weight loss was significantly greater than when a self-selected isocaloric diet was recommended. As the groups received similar treatment apart from the provision of the PPFs, the authors concluded that accurate portion control is an important factor in weight-loss success and that the use of packaged portion-controlled entrées is an effective method of achieving this.

Solid PPFs are a component of several commercial weight-loss programs. These programs include multiple components such as advice about nutrition, behavior and physical activity so it is not possible to isolate the influence of PPFs. It is likely, however, that the provision of PPFs contributed to improved weight loss and weight loss maintenance over 2 years in a RCT that compared a structured weight-loss program that included free preportioned prepared meals to usual care.^80^ Several other RCTs conducted in the context of commercial weight-loss programs found that weight loss was greater over 6 months^81^ or a year^82^ when PPFs were included than with a self-selected diet aimed at achieving the same reduction in energy intake. The 6-month study by Foster *et al.*,^81^ in particular, sought to isolate the effects of the portion-controlled diet from other components of the weight-loss intervention by keeping the other variables similar across the two treatment groups. The PPF group was provided with three entrées and one snack daily which they could supplement with conventional foods according to their program. After 6 months, the PPF group lost 7.3 kg and the control group lost 2.2 kg. The authors concluded that portion-controlled foods, whether as liquid shakes, meal bars or prepared servings of conventional foods, are beneficial and induce significantly greater weight loss than advice to consume an isocaloric diet that is entirely self-selected.

Wing^83^ suggests that making it clear exactly what should be eaten is a crucial component of the preportioned meal-provision strategy. Indeed, she found that giving patients detailed meal plans and shopping lists was as effective as providing preportioned food. She speculates that people need help with learning new eating habits, and provision of foods or eating plans facilitates this learning. Thus providing PPFs can be useful for weight management, but it is not clear whether improved weight loss is related to portion control or to the structure that food provision gives to the eating environment.^75^

### Does it matter when or how often PPFs are consumed or what they contain?

The European Food Safety Authority gives specific advice for the use of meal replacements and PPFs. Such products may contain a maximum of 250 kcal, and for weight loss two meals a day should be replaced while for weight loss maintenance one meal a day should be replaced.^69^ Such recommendations are based on the totality of the evidence rather than on studies designed to specifically optimize the effectiveness of portion-controlled meals. In particular, for PPFs it is not clear whether there is an optimal energy content or the number or type of meals that should be replaced.

Some commercial weight-loss programs start clients with PPFs across the entire day with the option of eating additional nutrient-rich, low-energy-dense foods, such as fruits and vegetables.^80, 81, 82^ Over time, clients transition to less frequent use, but they can continue to substitute PPFs for meals or snacks as desired. Although there are no known studies directly comparing different daily frequencies of consumption, PPFs have been shown to provide a useful tool if consumed twice a day or just at one meal. When PPFs consisting of frozen entrées were used twice a day, at lunch and dinner, they facilitated weight loss over 8 weeks.^78,79^ In another study, consumption of portion-controlled ready-to-eat breakfast cereal for breakfast and one other meal each day for 2 weeks was associated with reduced energy intake and improved weight loss compared with a group receiving no intervention.^84^ During an 8-week behavioral weight-loss intervention, the provision of a variety of portion-controlled foods just at breakfast reduced daily energy intake compared with non-portioned packages of the same foods but did not lead to greater weight loss.^85^ When subjects in a lab-based study chose a PPF for lunch, they ate around 250 kcal less each day than when they ate lunch from a buffet. This reduction was sustained over the 2-week intervention and led to greater weight loss.^86^ Thus substitution of one or two meals a day with PPFs can help people manage their energy intake. Although the ready availability, variety and convenience of PPFs makes them a useful tool to help consumers avoid the overconsumption associated with large portions, studies are needed to ascertain how to best incorporate them into diets to optimize their effectiveness.

It is possible that if PPFs are substituted for multiple meals across the day, or if they are very low in energy content, consumers may feel hungry or deprived and be at risk of succumbing to the appeal of the many other foods that are available. In the studies by Hannum *et al.*,^78,79^ the researchers speculate that they found a greater weight loss with the consumption of two frozen entrées a day in women than in men, because for the men the prescribed PPFs were only 43% of daily intake while for women they were 54%. Although there are no systematic studies to indicate what proportion of the diet should be provided by portion-controlled foods in order to maximize their effectiveness for weight loss, a recent study tested the influence of the energy content and energy density of PPFs on daily energy intake. Over a day, we systematically varied the energy content and energy density of compulsory preportioned entrées designed to provide approximately half of daily energy intake in order to determine the effect on intake of other discretionary foods.^87^ We found that, in non-dieting men and women, reducing both the energy content and the energy density of the entrées led to decreases in daily energy intake when a variety of other foods could be consumed *ad libitum*. Furthermore, decreasing the energy density allowed the portion size of the entrées to be maintained even while the energy content was reduced.^87^ More systematic investigations are needed to characterize the attributes of PPFs that most effectively control hunger, reduce energy intake, promote weight loss and lead to sustained use that will help with weight maintenance.

In summary, there is compelling evidence that consumption of liquid and solid PPFs at least once or twice a day can facilitate weight loss; however, it is not known if their use helps consumers understand what constitutes appropriate portions of different types of foods. It is also not clear whether there is an optimal composition or energy content of these foods that facilitates a reduction in daily energy intake.

## Beyond the ‘eat less' message

Although portion control is important for weight management, urging people simply to ‘eat less' of everything is unlikely to be the best approach to reduce intake. A more effective strategy may be to encourage greater consumption of foods low in energy density while managing portions of high-energy-dense foods. If people lower the energy density of their diet, they will be able to eat satisfying amounts of food while managing their body weight.

Data from several RCTs in adults support this approach. In one trial, overweight men and women were provided with isocaloric portions of either a low- or high-energy-dense food to be incorporated daily into a reduced-energy diet.^88^ A reduction in overall dietary energy density was the main predictor of weight loss during the first 2 months of the study. After 1 year, weight loss in the group incorporating two servings a day of low-energy-dense soup was 50% greater than in the group incorporating two servings of high-energy-dense dry snacks (7.2 vs 4.8 kg).^88^

In a second RCT, the effect of two strategies to reduce the energy density of the diet on weight loss in obese women was tested.^44^ One group was counseled to eat more water-rich foods, such as fruits and vegetables, and to reduce dietary fat. A comparison group was counseled to restrict portions and to reduce dietary fat. Both groups reduced the energy density of their diets, and both groups lost weight; however, after 12 months, the group counseled to eat more fruits and vegetables had a greater reduction in the energy density of their diet and lost 23% more weight (7.9 vs 6.4 kg) than the group told to reduce fat and restrict portions. Over the course of the year, participants in the lower-energy-dense diet group consumed 25% more food by weight based on their food records, had less hunger and felt greater satisfaction with the diet than those in the comparison group.^44^

In a third multicenter trial comparing three weight-loss strategies, across all participants the decrease in body weight over the first 6 months was related to the change in dietary energy density.^89^ The participants with the largest reduction in energy density lost more weight (5.9 kg) than those with the lowest change (2.4 kg). Of particular interest was that participants whose diet records indicated their diets were the lowest in energy density also reported eating the greatest weight of food (300 g more per day).^89^

These RCTs indicate that when dietary energy density was reduced, participants ate more food by weight. This was related to increased consumption of low-energy-dense foods, such as vegetables and fruits. These data reinforce the suggestion that dietary advice should focus on limiting portions of foods high in energy density and increasing portions of foods low in energy density, rather than limiting portions of all foods.

## Conclusions

In an obesogenic environment where large portions of energy-dense foods are pervasive and viewed as appropriate, it is challenging to find effective strategies to help people consume portions that match their energy requirements. Although there are a number of tools to teach people to recognize appropriate portions, it is not clear that these tools produce sustained changes in eating behavior that facilitate weight management. Another approach is to constrain the food environment through the use of preportioned items, whether liquid meal replacements or conventional foods. A number of studies demonstrate that substituting PPFs for one or more meals a day leads to greater weight loss than a self-selected diet. More studies are needed, however, to determine whether their use will lead to better understanding of appropriate portions and whether their consumption will be continued after the intervention has ended so that they facilitate long-term weight management. Understanding of appropriate portions must include the influence that energy density has on portions that can be consumed for a given number of calories. For weight management, people need to learn that they can eat a satisfying amount of food if they eat larger portions of low-energy-dense foods while limiting their portions of foods high in energy density.

## Notes

BJR's Volumetrics trademark is licensed exclusively by Jenny Craig, Inc.
